# On the Fractionation and Physicochemical Characterisation of Self-Assembled Chitosan–DNA Polyelectrolyte Complexes

**DOI:** 10.3390/polym15092115

**Published:** 2023-04-28

**Authors:** Ayesha Sajid, Matteo Castronovo, Francisco M. Goycoolea

**Affiliations:** School of Food Science and Nutrition, University of Leeds, Leeds LS2 9JT, UK

**Keywords:** chitosan, DNA, polyelectrolyte complex, AF4, SEC

## Abstract

Chitosan is extensively studied as a carrier for gene delivery and is an attractive non-viral gene vector owing to its polycationic, biodegradable, and biocompatible nature. Thus, it is essential to understand the chemistry of self-assembled chitosan–DNA complexation and their structural and functional properties, enabling the formation of an effective non-viral gene delivery system. In this study, two parent chitosans (samples NAS-032 and NAS-075; Mw range ~118–164 kDa) and their depolymerised derivatives (deploy nas-032 and deploy nas-075; Mw range 6–14 kDa) with degrees of acetylation 43.4 and 4.7%, respectively, were used to form polyelectrolyte complexes (PECs) with DNA at varying [–NH_3_^+^]/[–PO_4_^−^] (N/P) molar charge ratios. We investigated the formation of the PECs using ζ-potential, asymmetric flow field-flow fractionation (AF4) coupled with multiangle light scattering (MALS), refractive index (RI), ultraviolet (UV) and dynamic light scattering (DLS) detectors, and TEM imaging. PEC formation was confirmed by ζ-potential measurements that shifted from negative to positive values at N/P ratio ~2. The radius of gyration (*R_g_*) was determined for the eluting fractions by AF4-MALS-RI-UV, while the corresponding hydrodynamic radius (*R_h_*), by the DLS data. We studied the influence of different cross-flow rates on AF4 elution patterns for PECs obtained at N/P ratios 5, 10, and 20. The determined rho shape factor (*ρ* = *R_g_*/*R_h_*) values for the various PECs corresponded with a sphere morphology (*ρ* ~0.77–0.85), which was consistent with TEM images. The results of this study represent a further step towards the characterisation of chitosan–DNA PECs by the use of multi-detection AF4 as an important tool to fractionate and infer aspects of their morphology.

## 1. Introduction

Liquid–liquid phase separation obtained by mixing oppositely charged polyelectrolyte solutions through self-assembly or electrostatic interactions results in coacervation. These coacervates have been investigated as platforms for encapsulating and delivering biomolecules [1]. The choice of cationic polymers and lipidic dendrimers for non-viral gene delivery is limited due to their cytotoxic nature, with the exception of chitosan. Chitosan-based coacervates have been reported in the literature for their biomedical applications [2,3]. Chitosan is a binary linear copolymer of glucosamine (GlcN) and N-acetyl-glucosamine (GlcNAc) interconnected by β (1,4) glycosidic linkages. On an industrial and lab scale, chitosan is conventionally obtained from chitin deacetylation. Chitosan is a versatile biopolymer that has been applied in drug delivery, biomaterials, bioengineering, and gene therapy, as well as in several other sectors (agriculture, textiles, food, water). It is widely regarded as an effective and safe cationic carrier due to its high biocompatibility, biodegradability, mucoadhesion, and non-toxicity [4,5,6]. Chitosan-based carriers have been thoroughly researched as a non-viral delivery vector for the transport of nucleic acids such as plasmid DNA (pDNA), micro RNAs, small interfering RNAs (siRNA), and oligonucleotides, owing to its cationic property [7,8,9].

The amine groups in GlcN residues attain a positive charge at pH below ~6.0 [10]. The polyelectrolyte nature of chitosan allows it to bind to negatively charged DNA. Electrostatic interactions between the polycation chitosan and DNA solutions can yield the spontaneous formation of electrostatic self-assembled polyelectrolyte complexes (PECs) nanostructures, also denoted here as polyplexes or nanocomplexes, using a complex coacervation process [11,12,13]. The presence of the coulombic interactions between the oppositely charged polymers leads to the ionic condensation of the DNA during PEC formation and protection from nuclease degradation [10]. In the case of neutral or alkaline conditions, studies have shown that secondary non-electrostatic interaction (hydrogen bonding and hydrophobic interactions) can also be at play in the binding process between DNA and chitosan [14].

Both chitosan and DNA structural and chemical properties greatly influence the complexation and its underpinning phenomena. Among these, the stoichiometry of charge compensation, conformational adaptation of the two polyelectrolytes, kinetics, chitosan’s degree and pattern of acetylation, DNA’s sequence and conformation (single or double-stranded), and both polymers’ Mw (i.e., degree of polymerisation), are known to determine the PEC formation and structure. The formation conditions also dictate the complexation behaviour. The mixing ratio of positive to negative charges ratio (i.e., [–NH_3_^+^]/[–PO_4_^−^]–denoted for brevity as “N/P ratio”-), the total polymer concentration, pH, ionic strength, temperature, and mixing conditions, are known to be at play [15,16]. Of note, PECs formed by spontaneous electrostatic self-assembly are known to be under kinetic control and can undergo structural reorganisation with time [17]. Thus, it is paramount to have a comprehensive understanding of these intrinsic and extrinsic parameters [18]. Various reports have investigated different factors that can affect the transfection efficacy, stability, and particle size of chitosan–DNA nanocomplexes [19,20,21,22,23]. Understanding the influence of the polyplexes’ structure and morphology on the transfection efficacy entails the thorough characterisation of physicochemical and biophysical properties of these systems, with attention to the role of chitosan’s Mw and DA during DNA complexation. The physicochemical and biophysical characterisation of chitosan–DNA polyelectrolyte nanocomplexes is accessible via different conventional techniques such as optical density measurements, viscometry, dynamic light scattering (DLS), microscopic techniques, or ultracentrifugation. However, these techniques are limited in their scope, including interaction with the stationary phase, low selectivity and specificity and preservation of the structural integrity of the sample during analysis, among other. The lack of robust and well-assessed separation and characterisation techniques for the quality analysis of polyelectrolyte complexes can limit their applications [24].

Asymmetric flow field flow fractionation (AF4) is a polymers/macromolecules and nanoparticles separation and characterisation technique which is coupled with multi-detection by multi-angle laser light scattering (MALS), differential refractive index (RI), UV-Vis, dynamic light scattering (DLS), and other types of more specialised detectors. AF4 is used to characterise the molecular weight and size distribution as well as the shape and conformation of both synthetic and natural polymers (e.g., polysaccharides and DNA), biological macromolecules (e.g., proteins, mucin, antibodies, nanobodies), viruses, cellular vesicles, as well as polymeric and metallic nanoparticles, and nano- and micro-gels [25,26,27,28]. AF4 separation is based on field flow fractionation principle introduced by Giddings and Wahlund [29,30]. The sample separation takes place in a narrow ribbon-like channel. The upper wall of the channel is a solid impermeable plate, while the lower one is composed of a permeable porous steel frit with a trapezoidal spacer and ultrafiltration membrane placed in the middle. The bottom plate and the filtration membrane form the accumulation wall. Once the sample enters the channels, it is pushed down towards the accumulation wall under the external force going downwards, which is called the cross-flow. At the same time, the analytes diffuse upwards to the centre of the channel due to the Brownian motion, and the particles separate into different laminar flows owing to their varying diffusion coefficient (*D*). Thus, a steady equilibrium layer establishes under two opposing forces, causing the highest concentration of the sample to accumulate near the wall, and it is reduced in the centre. The velocity of the flow inside the channel is parabolic (the largest velocity in the centre and gradually decreases to both sides), and the particles will travel based on their distance from the wall. Smaller particles near the centre line will elute earlier than larger ones due to their higher diffusion coefficient (normal elution mode). In AF4, the retention time (*t_r_*) is inversely proportional to *D*, given by Equation (1) below:

(1) $$t_{r}=\frac{t^{0}V_{x}\omega^{2}}{6V^{0}}\frac{1}{D}$$

where *t*^0^ is the retention time of the void peak (void time), *V_x_* is the cross-flow rate, *ω* is the channel thickness, and *V*^0^ is the channel void peak volume [30]. 

Several key factors are critical to achieving a good fractionation quality. These include the channel height, sample focusing time, mobile phase, type of analytes and filtration membrane, injected volume/concentration, and the cross-flow rate [31]. By contrast, with size exclusion chromatography (SEC), AF4 eliminates the use of stationary phase, reduces shear force, and offers better separation over a wide range of sizes, thus eliminating the drawbacks of SEC [26,27,28,29,30,31,32]. As with SEC, AF4 is typically attached to online multiple downstream detectors such as MALS, RI, UV-Vis, and DLS [33]. AF4 is regarded as a multi-dimensional platform for improved and accurate size analysis of polymers and nanomaterials. The feasibility of AF4 to fractionate and characterise chitosan–DNA complexes has been demonstrated [34,35], in particular, to study the size, distribution, composition, and conformation. However, AF4 is still a relatively minimally explored technique for the characterisation of PECs, including chitosan–DNA nanocomplexes.

The present work involves the formation and characterisation of chitosan and DNA polyelectrolyte complexes and the study of the influence of different degree of acetylation and molecular weights of chitosan on the physicochemical properties of DNA/chitosan complexes. To this end, we characterised the formed PECs by AF4-MALS-RI-UV-Vis-DLS and evaluated the impact of the cross-flow rate on the overall fractionation and particle size distribution. We also imaged the formed PECs by TEM. The combined *R_h_* and *R_g_* data from DLS and MALS, respectively, along with the TEM images, enabled us to gain insight into the details on the size and morphology of the chitosan/DNA complexes.

## 2. Materials and Methods

### 2.1. Materials

High-purity biomedical-grade chitosan samples were purchased from FlexiChem (Uttran, Stockholm, Sweden) (code: Viscosan, Batch No. NAS-032 and NAS-075). N-acetylglucosamine (CAS: 7512-17-6, used as a standard for spectrophotometric degree of acetylation determination), single-stranded calf thymus DNA (ssDNA ≥ 65% with the base composition of 41.9 mole% G-C content and 58.1 mole% A-T, Cat No. D8899), sodium acetate trihydrate (CAS: 6131-90-4), sodium nitrite (CAS: 7632-00-0), acetone (CAS: 67-64-1) and 0.1 M HCl (Titripur, CAS: 7647-01-0) were purchased from Merck Life Science (Gillingham, UK). Ammonia solution (CAS: 1336-21-6) and acetic acid glacial (CAS: 64-19-7) were purchased from Fisher Scientific (Loughborough, UK). Milli-Q water (18.3 MΩ.cm) was used throughout.

### 2.2. Depolymerisation of Chitosan

Parent chitosans (NAS-032 and NAS-075) were depolymerised by an in situ nitrous acid reaction formed by the dissolution of sodium nitrite to generate lower molecular weight chitosan oligomers (hereafter referred to as deploy nas-032 and deploy nas-075) as per the protocols developed by Allan et al. [36,37]. Briefly, chitosan was dissolved in 20% stoichiometric excess of acetic acid at a concentration of 4 mg/mL. The amount of sodium nitrite required for the reaction was 0.35 and 0.40 mg/mL for deploy nas-032 and deploy nas-075, respectively. Upon the addition of sodium nitrite, the reaction was carried out at room temperature for 21 h under magnetic stirring in a dark room. The depolymerised chitosan was then precipitated out by increasing the pH with ammonia (pH > 8) and pure acetone was used to enhance the precipitation of the depolymerised chitosan. The precipitated chitosan was separated using high-speed centrifuge (Beckman Coulter Avanti J-30I) at 10,000 RPM for 5 min and washed multiple times using different acetone/water ratios (*v*/*v* = 80/20, 90/10, 100/0). Finally, the precipitate was transferred to a glass petri dish for drying [36,37].

### 2.3. Characterisation of Chitosan: Degree of Acetylation and Molecular Weight Distribution

The degree of acetylation of the parent chitosan samples (NAS-032 and NAS-075) was determined through UV spectrophotometry (Specord 210 plus UV/Vis spectrophotometer) using quartz cuvettes as per the improved method reported by Pedroni et al. (2003) [38]. Briefly, standard solutions of N-acetyl glucosamine (GlcNAc) were prepared at a concentration from 0.01–0.08 mg/mL in 0.1 M of HCl. Chitosan samples NAS-032 and NAS-075 were dissolved in the same solvent at a concentration of 0.1 and 0.3 mg/mL, respectively. All spectra were recorded at λ = 201 nm and a calibration curve of absorbance against the N-acetyl glucosamine concentration was drawn. The molecular weight distribution characterisation of parent chitosans and their depolymerised derivatives was determined using an AF2000 Multiflow system from Postnova Analytics (Postnova Analytics GmbH, Landsberg, Germany), which was set to operate in SEC and AF4 modes using the protocols reported in a recent study of our group [39]. In SEC mode, we used three columns (TSK gel G6000PW, G5000PW, and G2500PW) for the analysis of parent chitosan (NAS-032 and NAS-075), while for depolymerised chitosan (deploy nas-032 and nas-75), we used TSK gel G5000PW, G3000PW, and G2000PW columns from Tosoh Bioscience, along with a guard column at a temperature of 30 °C. Chitosan samples were prepared at a concentration of 1 mg/mL by dissolving overnight in solvent (0.3 M acetic acid/0.2 M sodium acetate, pH 4.5) and filtered through a 0.22 µm regenerated cellulose (RC) syringe filter (Bio Vision). The same solvent was used as the mobile phase after filtration through 0.1 µm mixed cellulose esters (MCE) membrane filter.

In AF4 mode, the samples were dissolved in acetate buffer (0.02 M sodium acetate/0.18 M acetic acid with a pH of 3.7) at a concentration of 2 mg/mL and filtered through 5.0 µm MCE membrane filter. The flow conditions were set with a cross-flow of 3 mL/min and programmed with time delay exponential decay for optimal characterisation of samples. The refractive index increment (dn/dc) for chitosan was set at 0.19 mL/g [40]. In both separation modes, the system was coupled with multi-angle light scattering (MALS) and differential refractive index (RI) detectors (Postnova). The data were processed using the Nova SEC and NovaFFF AF2000 software for SEC and AF4, respectively.

### 2.4. Formation of Polyelectrolyte Complexes

Chitosan solutions were prepared by stoichiometrically dissolving with 0.1 M HCl, based on their respective degree of acetylation in Milli-Q water at the concentration of 1 mg/mL. The DNA solution was prepared (0.03 mg/mL) in water. Chitosan–DNA polyplexes were prepared at varying N/P ratios (0.1, 0.5, 1, 2, 5, 10, and 20). For complexation, a given volume of chitosan (calculated based on its equivalent amine content) was added to 1 mL of DNA and stirred for a few minutes, as per the general protocol reported elsewhere [41].

### 2.5. Characterisation of Chitosan–DNA Polyplexes

#### 2.5.1. Zeta (ζ)-Potential Measurements

ζ-Potential measurements of the chitosan–DNA polyplexes of varying N/P ratios were carried out with Malvern Zetasizer Ultra ZSU5700 (Malvern Panalytical Ltd., Malvern, UK) at 25 °C equipped with a 4 mW He/Ne laser beam (λ = 633 nm) output and using Doppler velocimetry applying phase analysis light scattering (M3-PALS). To this end, analytes were filled into a folded capillary zeta cell (DTS1070, Malvern) and the Smoluchowski approximation was used to determine the ζ-potential of the nanocomplexes. Measurements were done in triplicate. 

#### 2.5.2. Batch-Mode Non-Invasive Back Scattering—Dynamic Light Scattering (NIBS-DLS) 

Batch-mode hyrodynamic radius (*R_h_*) measurements of depolymerised chitosan (deploy nas-032 and nas-075) and calf thymus DNA polyplexes of varying N/P ratios were carried out at 25 °C by non-invasive back scattering dynamic light scattering (NIBS-DLS) at 173° using the same instrument as for ζ-potential measurements. The Zetasizer XPLORER software (version 3.2.1) was set to auto mode to compute the Z-average *R_h_* values using the Stokes–Einstein relationship.

#### 2.5.3. Asymmetric Flow Field Flow Fractionation (AF4)

Nanocomplexes were fractionated and characterised by AF4 with multi-detection. Briefly, the measurements were performed using a Postnova Analytics AF2000 Multiflow system (Landsberg, Germany). The system was operated in AF4 mode and coupled with multi-angle light scattering detector (21 angles, PN3621), a dual UV detector (λ = 220 and 280 nm, PN3211) and a refractive index detector (PN3250). The system had an analytical AF4 asymmetric channel (Postnova Z-AF4-CHA-611) with a spacer of 350 µm. The temperature was controlled using a thermostat (PN4020) at 30 °C and the membrane used was a regenerated cellulose membrane with a cutoff 10 kDa (Z-AF4-MEM-612-10 kDa). In addition to these detectors, an online DLS Zetasizer instrument (Nanoseries, Malvern) was also used in flow mode. The mobile phase used for these experiments was acetate buffer (0.02 M sodium acetate, 0.18 M acetic acid with a pH of 3.7 filtered through 0.1 µm, similar to the one used in the protocol established by our group to characterise the Mw of chitosan [39] (Section 2.3).

**Figure 1:**
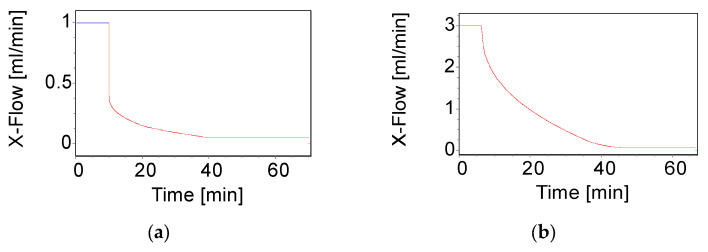
Graphical representation of the cross-flow (CF) profiles programmed with time exponential decay. (**a**) Cross-flow: 1 mL/min; (**b**) Cross-flow: 3 mL/min.

The chitosan (NAS-032, NAS-075, deploy nas-032, and deploy nas-075)–calf thymus DNA nanocomplexes were prepared as mentioned in Section 2.4. However, to perform AF4 measurement, the stock solutions of both chitosan and DNA were filtered before complexation through a 5.0 µm mixed cellulose ester (MCE) membrane filter. Two elution protocols differing in their flow conditions and cross-flows (i.e., 1 and 3 mL/min) were used to analyse these complexes except for polyplexes formed by depolymerised chitosan, in which case only method 2 (as shown in Table 1) was utilised. For each run, 50 µL (for polyplexes comprised by NAS-032 and NAS-075 chitosans) and 100 µL (for polyplexes comprised by deploy nas-032 and deploy nas-075 chitosans) of the sample was injected. The elution was carried out using a cross-flow rate programmed with an exponential-linear decay (for 1 mL/min) and a time delay-exponential decay for 3 mL/min (Figure 1, Table 1). The data were processed by NovaFFF AF2000 software to obtain the Mw distribution parameters. 

### 2.6. Negative Staining Transmission Electron Microscopy (TEM)

For the morphological investigation of the NAS-032 and NAS-075 DNA nanocomplexes, TEM imaging was carried out using a FEI-Tecnai G^2^-Spirit electron microscope operating at a voltage of 120 keV equipped with a tungsten filament. Samples were prepared by dropwise addition of 5 µL of the chitosan–DNA polyplexes onto a glow discharged (using PELCO easy Glow discharge unit), carbon-coated 400 mesh copper grids. The samples were then negatively stained with 2% uranyl acetate solution using the side blot method. The images were captured using Gatan Ultrascan 4000CCD camera and processed through Digital Micrograph software.

## 3. Results and Discussion

### 3.1. Spectrophotometric Determination of the Degree of N-Acetylation (DA) of Parent Chitosan

Chitosan, obtained at industrial scale by thermoalkaline deacetylation of chitin, contains two far ultraviolet chromogenic groups, N-acetyl glucosamine (GlcNAc) and glucosamine (GlcN), showing no interaction within the polymer that would affect its UV absorption. Chitosan’s DA is defined as the molar proportion of GlcNac units to the total of GlcNAc and GlcN in chitosan, and it is a parameter that influences the polymer’s physical, chemical, and biological properties. It also determines the charge density and, therefore, is a critical factor for chitosan–DNA electrostatic complexation.

Many techniques have been proposed to determine chitosan’s DA, such as potentiometric and conductimetric titration, ^1^H NMR or FTIR spectroscopy, and elemental analysis [42,43]. A relatively straightforward method based on UV spectroscopy was originally developed by Muzzarelli et al. [44]. Variations of this protocol, by using dual standards [45] or improved first derivative UV [46], have been proposed. In our study, we used a single GlcNAc standard calibration curve prepared in 0.1 M HCl as per the method of Pedroni et al. [38]. The DA values determined experimentally and reported by the manufacturer are shown in Table 2. Both values can be regarded in close correspondence within an experimental error of ~10%. In subsequent calculations, we used the experimentally determined DA values. Of note, the NAS-075 chitosan’s DA was very low (<~5%), characteristic of a nearly homopolymeric chain of polyglucosamine.

### 3.2. Molecular Weight Distribution of Chitosan 

**Figure 2:**
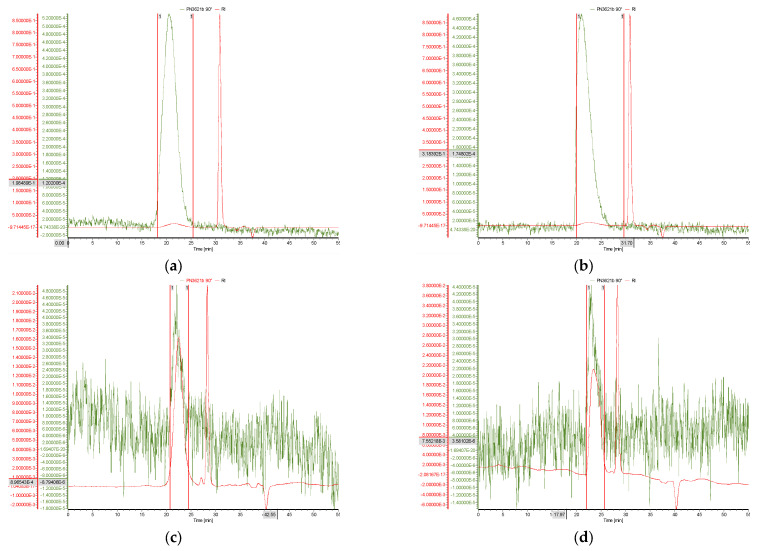
SEC-MALS-DRI chromatographs for: (**a**) NAS-032 (**b**) NAS-075, (**c**) Deploy nas-032 and (**d**) Deploy nas-075; green trace is MALS 90° signal (set as baseline for the other 20 scattering angles), red trace is RI signal. The mobile phase was 0.3 M CH_3_COOH/0.2 M C_2_H_3_NaO_2_, pH = 4.5.

The coupling of fractionation techniques like SEC and AF4 to MALS and RI detectors provides a powerful tool to characterise polymers. In the present study, we used both methods to characterise the parent and depolymerised chitosan samples fully. Representative SEC-MALS-RI chromatogram results for the chitosan samples used in this work are shown in Figure 2. Two high-purity biomedical grade parent chitosans (NAS-032 and NAS-075) were used to generate their low molecular weight derivatives (deploy nas-032 and deploy nas-075) by controlled acid depolymerisation. To this end, nitrous acid was used in a homogenous reaction in which the amount of nitrous acid used is stoichiometric to the number of glycosidic bond breakage [47]. In short, the depolymerisation mechanism includes the reaction of the in situ-generated nitrous acid with the amine group of the glucosamine (GlcN) units, which leads to the breaking of glycosidic linkage and formation of 2,5-anhydro-D-mannofuranose at the reducing end [36,37]. 

An inspection of the chromatograms of the parent chitosan samples shown in Figure 2a,b shows one well-defined predominant peak on the MALS 90° signal and two peaks for the concentration detector (RI) trace. In detail, these are a tiny RI peak at retention time (*t_r_*) of ~21.5 min that corresponds closely with the MALS 90°, and a sharp high-intensity second RI peak centred at *t_r_* ~31 min with no accompanying MALS 90° signal. Both peaks are also noticeable on the chromatograms of the depolymerised chitosans shown in Figure 2c,d. However, for these samples, a highly noisy MALS 90° trace with a peak centred at *t_r_* ~22.5 min is concurrent with a well-defined intense RI peak. As for the parent chitosans, a second RI peak centred at *t_r_* ~31 min is present without having a corresponding MALS 90° signal for none of the chromatograms. 

When compared the MALS 90° SEC elution curves of the parent and depolymerised chitosans, only marginal differences in their *t_r_* values are apparent despite the large difference in their molecular weights (Figure S1). However, a detailed data analysis (not shown) confirmed a high correlation (R^2^ = 0.929) between the log *t_r_* vs. log Mw, as expected in SEC polymers separation. The MALS 90° signals for the parent chitosans were substantially more intense and smoother than those of the depolymerised counterparts (Figure S1). This is the expected consequence of the greater light scattering expected for the high molecular weight polymer species as compared to the depolymerised fractions that showed much noisier MALS 90° signals. These results are in good agreement with those of Affes et al. [48]. The peaks of MALS 90° and RI with *t_r_* ~21.5 min were used to define the regions of integration (denoted by the red lines) and used to the determine the molecular weight distribution of the samples. In turn, the second RI peak (*t_r_* ~31 min) was attributed to the mobile phase (Figure S4), thus signalling the completion of the elution of the exclusion volume.

**Figure 3:**
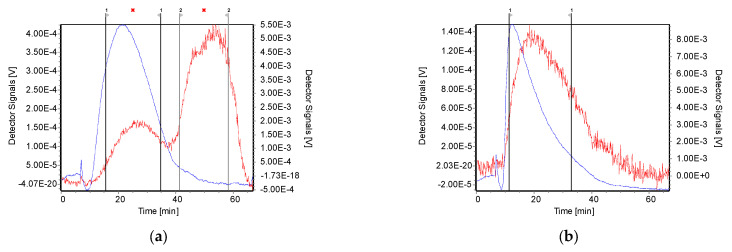

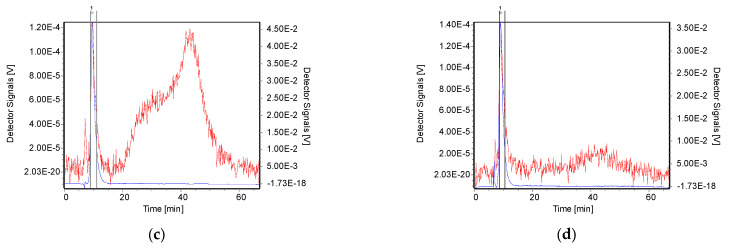
Fractograms obtained from AF4-MALDS-RI of (**a**) NAS-032, (**b**) NAS-075, (**c**) Deploy nas-032, and (**d**) Deploy nas-075. Red trace is MALS 90° signal, blue trace is RI signal. The mobile phase was 0.18 M acetic acid/0.02 M sodium actetate (pH = 3.7) and the cross-flow = 3 mL/min.

In contrast with the Mw distribution analysis conducted by SEC on the samples that were filtered through 0.22 µm RC membranes, the samples were also analysed by AF4 and in this case, filtered through a much larger pore size (5.0 µm) MCE membranes than for SEC analysis. Figure 3 shows AF4 fractograms overlaying the light scattering (MALS 90°) and RI signals for the four samples. For chitosan NAS-032 (Figure 3a), two MALS 90° and one RI peaks were resolved. The main MALS 90° peak (fractions eluting between 12 and 37 min) represents 98% of the total recovered mass. 

As the elution proceeds, this peak is fused with a second partially resolved broad and large peak centred at ~55 min that accompanies the tail of the RI signal. This second peak is attributed to the presence of supramolecular aggregates that are separated from the well-dissolved polymer, accounting for a small portion of the recovered sample. We have observed similar aggregates in AF4 fractograms of commercial chitosan samples [39] and locust bean gum [49], finding that they disappeared when they were filtered through 0.22 µm pore membranes. For deploy nas-032 (Figure 3c), note the sharp intense MALS 90° and concomitant RI peak eluting within 9–11 min (99% of recovered mass). A second MALS 90 ° peak, with no accompanying RI signal counterpart, appears as a broad band that attains a maximum intensity at elution time ~40 min and exhibits a prominent shoulder on the lower elution time side. When compared with the features of the second MALS 90° peak of the parent NAS-032 chitosan, it seems reasonable to suggest that the presence of this peak offers diagnostic evidence of the incomplete depolymerisation of the supramolecular aggregates present. This finding was unexpected and suggested that the nitrous acid hydrolysis reaction did not proceed homogeneously, but there was a greater preference for the dissolved unaggregated sample. By contrast, for chitosan NAS-075 (Figure 3b), only single broad and skewed MALS 90° and RI peaks were observed between ~10 and ~60 min. Additionally, the fractogram for the deploy nas-075 sample shows a sharp MALS 90° and RI peaks with maximum intensities centred at ~10 min and only a broad small MALS 90° peak at ~45 and ~60 min. In this case, we can be confident that the acid depolymerisation proceeded almost to completion on the entire sample. The fractograms of the NAS-032 and NAS-075 chitosan samples are in good keeping with results obtained in previous studies on commercial chitosan samples in our lab [39].

The molecular weight distributions of the chitosans used in this work determined through both SEC and AF4 are summarised in Table 3. Within experimental error, the results of both techniques exhibited reasonable agreement on the Mw distribution parameters determined by SEC and AF4 for the parent and depolymerised chitosans. Mw of the parent chitosan samples was within the same order of magnitude and their polydispersity (Ð ~2.0) was also very similar. However, for the depolymerised chitosans, the differences between Mw distributions obtained from the two techniques were more pronounced, especially for deploy nas-075 where the Mw varied by almost two-fold and showed a high polydispersity (Ð ~4.7). This could be due to the presence of multiple fractions of depolymerised sample obtained after chemical hydrolysis as suggested by the noisy MALS 90° SEC signal. The average Mw, determined from both techniques (SEC and AF4), confirmed the decrease in Mw from 153 to 9.3 kDa and 121 to 10 kDa for NAS-032 and NAS-075, respectively. The results concur with those of Augsten and Mader, who investigated the molar mass of various types of chitosan through AF4-LS and found effective separation capability over a broad molar mass range, absolute molar mass determination, and molecular structure parameters (molar mass-gyration radius relationship) [50]. The Mw of chitosan is a key parameter known to impact the particle size, stability of chitosan/DNA PECs, cellular uptake, and ultimately the intracellular dissociation of DNA from the complex [16]. Studies have shown that the size of PEC enlarges with the increase in the Mw of chitosan. Very low molecular weight chitosan is insufficient for stable nucleic acid complexation [51]. Alatorre-Meda et al. have reported that high molecular weight chitosan forms bulkier complexes with DNA and the dependence of *R_h_* is linear with Mw [52]. Thus, medium and high molecular weight chitosan are preferred to form DNA–chitosan complexes. Studies have shown that by using low molecular weight chitosan and oligomers for gene delivery vectors we can improve the transfection efficiency. A fine balance should be sought to achieve DNA compaction and protection (by high Mw chitosan) and effective gene delivery and unpackaging (by low Mw chitosan) [21,53]. 

The recovery percentage was calculated through the peak area measured using a concentration detector and the corresponding dn/dc value [54]. The recovery attained by SEC was higher than AF4. This loss in recovery in AF4 has been attributed to the membrane adsorption of the sample, which is more pronounced when a high cross-flow rate is applied, as shown in previous studies [39]. This, however, does not compromise the efficiency of the fractionation, nor of the data analysis outcomes. 

### 3.3. ζ-Potential of Polyelectrolyte Complexes

When mixed in an aqueous solution, oppositely charged polyelectrolytes, such as chitosan and DNA, associate spontaneously by electrostatic self-assembly, resulting in the formation of a polyelectrolyte complex whilst releasing counter-ions [55]. The molar ratio of glucosamine [–NH_3_^+^] to DNA phosphate [–PO_4_^−^], for brevity here denoted as the N/P ratio (corresponds to the mixing ratio of amine in chitosan vs. the phosphates in the nucleotide of DNA) is a significant factor that impacts the formation and stability of the nanocomplexes. It determines the stoichiometry of charges compensation during the complex formation. For instance, a DNA mass concentration of 0.03 mg/mL is equivalent to a [–PO_4_^−^] molar concentration of 7.9 × 10^−5^ mol/L, given that the average molar mass of a single nucleotide is 379 g/mol. Similarly, 1 mg/mL of chitosan in solution is equivalent to [–NH_3_^+^] molar concentration of 3.1 × 10^−3^ (for NAS-032) and 5.8 × 10^−3^ mol/L (for NAS-075), respectively, as determined through the following equation:

(2) $${nNH}_{2}=\frac{\left[mcs\times \left(1-\frac{DA}{100}\right)\right]}{M_{CS}}$$

where *nNH*_2_ is the molar concentration of glucosamine, *mcs* is the mass of chitosan in grams, and *M_CS_* is the average molar mass of chitosan.

**Figure 4:**
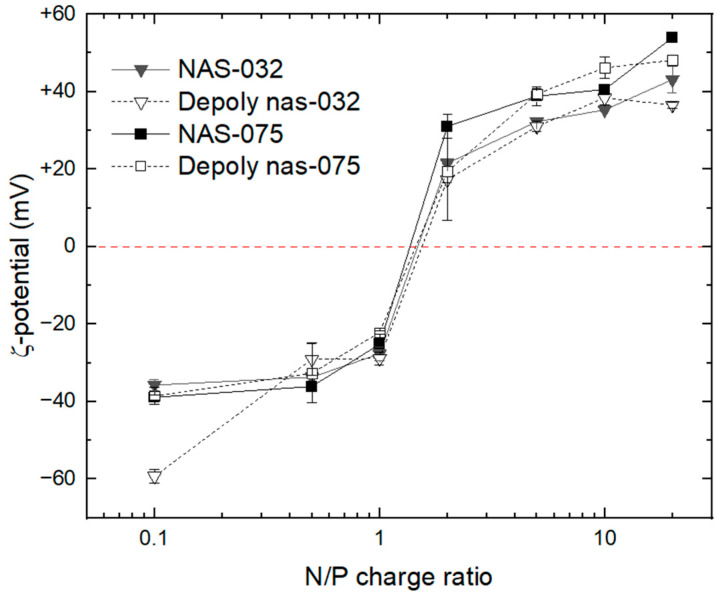
ζ-Potential results of Chitosan–DNA complexes as a function of their stoichiometric charge ratio (N/P 0.1, 0.5, 1, 2, 5, 10, and 20). The red dotted line indicates the zero level, where isoelectric condition is achieved.

We progressively increased the N/P ratio by adding a small volume of chitosan until complexation was achieved. The confirmation of complex formation was attained by ζ-potential results (Figure 4). The shift in the ζ-potential corresponds to the formation of complex between DNA and chitosan. The inversion of ζ-potential from negative to positive marks the isoelectric point of the polyplexes where the ratio between the negative charges of DNA is neutralised by the positive charges of chitosan, hence it marks the stoichiometry of full charge compensation during the complexation [56]. Beyond this point, the ζ-potential increased sharply up to N/P = 2. At higher ratios, this rise was moderate and tended towards constant value.

The results in Figure 4 shows that for the lower charge density of NAS-032 (DA ~43.4%) and deploy nas-032, both required a greater amount of chitosan than that of NAS-075 (DA ~4.7%) and deploy nas-075 to compensate for the equivalent number of charges of DNA during complex formation when prepared at identical stoichiometric N/P ratios. MacLaughlin et al. explored the ability of chitosan and its depolymerised oligomers to condense plasmid DNA and act as in vivo carriers. They used a similar approach to ours and found that the molecular mass of chitosan oligomer declined by increasing the amount of sodium nitrite used for depolymerisation, but the degree of acetylation (DA) remained unchanged [57]. It is known that the nitrous acid only attackes the amine group and not the N-acetyl moieties. Based on this, we were confident that the DA, hence charge density, of the depolymerised low molecular weight chitosan derivatives was similar to their parent counterparts, thus retaining their ability to complex with DNA after depolymerisation.

The cationic charge density of chitosan, depending on the degree of acetylation and pH of the medium, is a principal factor in forming complexes with nucleic acids [58,59]. Here, all chitosan samples presented an overall similar behaviour in complex formation despite having widely different DA and Mw. Indeed, we observed that the overall ζ-potential pattern of dependence on N/P ratio was not affected by the chitosan’s Mw, and invariably we observed a sigmoidal dependence in which the ζ-potential inverted from negative (~−35 mV) to positive (~+40 mV) for the complexes of N/P < 1.0 to N/P > 2.0, respectively. A closer inspection of N/P = 2.0, however, shows that the ζ-potential declined from 22 to 17 mV and 31 to 19 mV for deploy nas-032 and deploy nas-075 polyplexes, respectively, denoting a lower effective charge compensation for the depolymerised chitosans. This can be ascribed to a lower enthalpic gain due to loss of multivalency of the shorter polymer. Thus, for the same degree of acetylation, a low molecular weight chitosan requires a higher N/P charge ratio to compensate at the same effective charge stoichiometry than do high Mw chitosans [60]. Despite a somewhat lower ζ-potential of the polyplexes comprising low Mw chitosan, the complexation capacity of these oligomers does not seem to be affected by their size, and they retain their ability to associate with DNA completely. 

The optimum transfection in terms of size, ζ-potential and morphology of the polyelectrolyte complexes with dependence of chitosan (amine)-DNA (phosphate) charge ratios, have been reported which was mostly higher than the isoelectric point of the corresponding nanocomplexes [55,61,62]. Alatorre-Meda et al. investigated the impact of Mw of chitosan on polyplex formation at low pH and concluded that where the transfection ability of the complexes depends on the Mw, the ζ-potential is indicative of the electrostatic behaviour that remains the same [55]. The condensation of nucleic acid using cationic polymers to form compact structures is a crucial process in gene delivery. The net positive charge on the surface of a given non-viral gene delivery carrier is an important prerequisite for efficient gene delivery. A study by Valente et al. [63] showed that chitosan-based polyplexes exhibited a negative ζ-potential up to N/P ~1, and beyond this, a higher positive charge was accomplished. They also proposed that the value of the ζ-potential can suggest whether the negatively charged DNA is encapsulated in the core or localised on the surface of the polyplex. Thus, considering the values obtained in our study, we can assert that the fabricated polyelectrolyte complexes have a shell of the cationic polymer, and the nucleic acid is compacted inside this shell. 

### 3.4. AF4 Characterisation of PEC

Turning now to the experimental characterisation of the chitosan–DNA PECs, we also used AF4-MALS-RI-UV-Vis-DLS as discussed in Section 3.2, but leveraged its power to separate and characterise the size and conformation of colloidal nanoparticles. The carrier liquid used for these measurements was acetate buffer (pH ~3.7), which confers a positive charge to the surface of the filtration membrane (ζ-potential ~+10 mV), as documented in our previous studies [39]. In this regard, the mobile phase’s properties, like pH, composition, and ionic strength, are known to significantly influence the particles’ interaction with the filtration membrane’s surface, thus affecting the sample’s adsorption and retention time [64]. Optimising such properties can prevent the aggregation of the particles, reduce the chances of interaction between the sample and the membrane, and maximise sample recovery. Thus, selecting the carrier liquid compatible with the AF4 systems and its coupled detectors is of utmost importance. 

Initially, we characterised polyelectrolyte complexes formed using the parent chitosan samples. The fractogram of polyplexes formed at low N/P ratios (N/P 0.5, 1 and 2, results not shown) gave very noisy and distorted signals; thus, only nanocomplexes formed at high N/P charge ratios (N/P = 5, 10, and 20) were further studied by AF4. We reason that at low N/P ratios (i.e., low chitosan concentration), the negatively charged DNA can interact with the membrane. The elution profile of polyplexes at N/P = 2, was also not clear, despite the fact that it gives a positive ζ-potential. This could be because the injection volume used for AF4 separation of parent chitosan–DNA polyplexes was only 50 µL for each ratio, and under the field flow fractionation, the samples can be significantly diluted by the mobile phase which can limit the analysis of the low concentrated samples by detectors. For PECs obtained at higher N/P (i.e., upon increasing chitosan concentration), the increased ζ-potential favoured a better elution generated by the repulsive forces between the cellulose membrane and positively charged chitosan–DNA PECs. 

**Figure 5:**
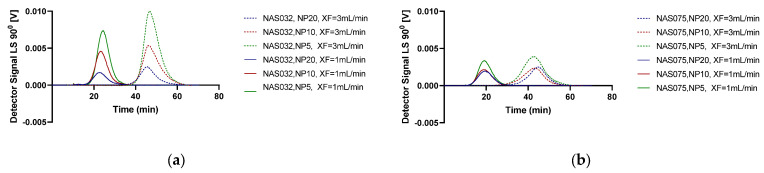
Light scattering 90° fractograms of (**a**) chitosan NAS-032-DNA and (**b**) chitosan NAS-075-DNA nanocomplexes formed at N/P ratio 5, 10, and 20 with different cross-flow (XF) rates of 1 and 3 mL/min (as shown in the figure legends).

**Figure 6:**
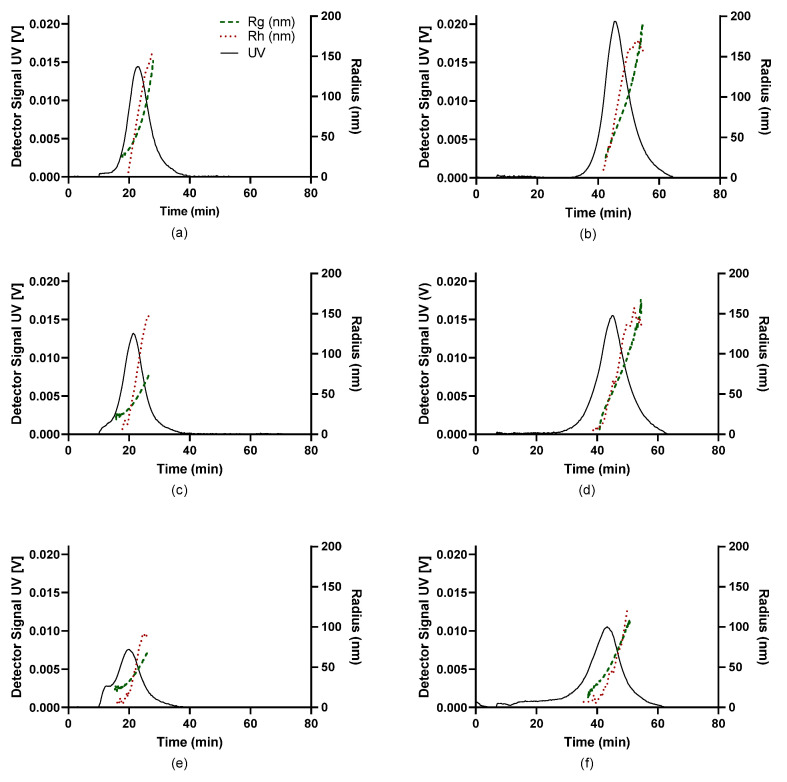
Fractograms for UV-Vis, *R_g_*, and *R_h_* (per legend) of NAS-032-DNA nanocomplexes obtained by asymmetric flow field-flow fractionation at cross-flow rate (XF) of 1 mL/min (**a**,**c**,**e**) and 3 mL/min (**b**,**d**,**f**). Black trace is UV signal (concentration), green dots is *R_g_*, red dots is *R_h_*. (**a**) NAS-032-DNA N/P 5, XF1; (**b**) NAS-032-DNA N/P 5, XF3; (**c**) NAS-032-DNA N/P 10, XF1; (**d**) NAS-032-DNA N/P 10, XF3; (**e**) NAS-032-DNA N/P 20, XF1; (**f**) NAS-032-DNA N/P 20, XF3.

**Figure 7:**
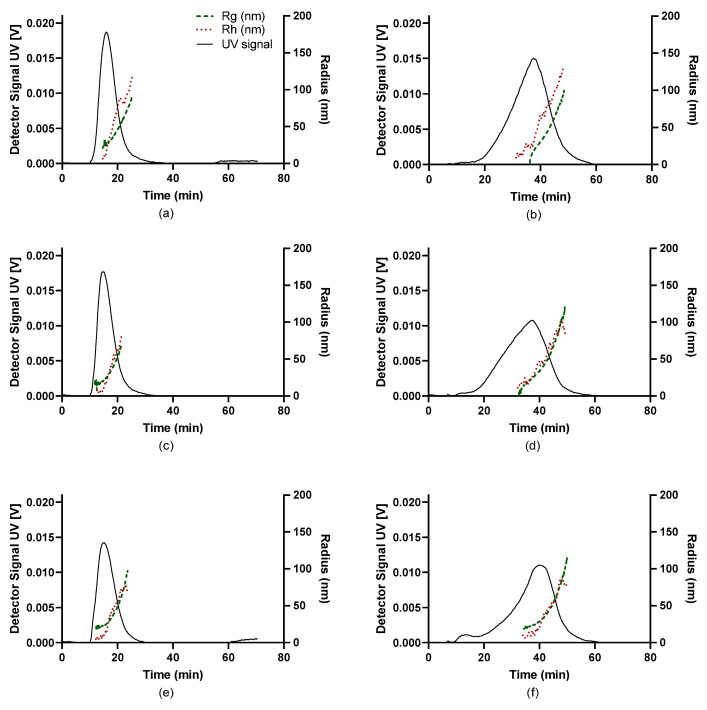
Fractograms for UV-Vis, *R_g_*, and *R_h_* (per legend) of NAS-075-DNA nanocomplexes obtained through asymmetric flow field-flow fractionation at cross-flow (XF) of 1 mL/min (**a**,**c**,**e**) and 3 mL/min (**b**,**d**,**f**). Black trace is UV signal (concentration), green dots are *R_g_*, red dots are *R_h_*. (**a**) NAS-075-DNA N/P 5, XF1; (**b**) NAS-075-DNA N/P 5, XF3; (**c**) NAS-075-DNA N/P 10, XF1; (**d**) NAS-075-DNA N/P 10, XF3; (**e**) NAS-075-DNA N/P 20, XF1; (**f**) NAS-075-DNA N/P 20, XF3.

To achieve the maximum separation efficiency of the PECs by AF4, we studied the influence of two different cross-flow rates (1 and 3 mL/min) on polyplexes formed by NAS-032 and NAS-075. The cross-flow is the driving force that counteracts the complexes’ Brownian motion, and the interplay between the two phenomena governs the separation efficiency of particles of varying sizes at different laminae of the channel [65]. In this regard, it is essential to optimise the cross-flow rate to achieve the best resolution. Studies have shown that a high cross-flow and long focusing time will lead to a greater probability of sample aggregation. If the cross-flow rate is too low, it will lead to poor separation, while if too high, it increases the risk of aggregation and membrane interaction. In addition, the decrease in sample recovery at a high cross-flow rate is because of forcing the particles closer to the channel membrane for longer times [28]. The results in Figure 5 depict the MALS 90° signal fractograms for the studied nanocomplexes at varying N/P ratios and at the two studied cross-flow rates. As expected, the elution (retention) time shifted from ~10–30 to ~30–60 min when the cross-flow rate increased from 1 to 3 mL/min, respectively. This is explained by the direct relationship between the retention time and the cross-flow rate (Equation (1)). A closer inspection of Figure 5a,b also shows that the overall intensity and area under the curve of the MALS 90° peaks at the two cross-flow rates decreased as the N/P ratio increased (N/P 5 > N/P 10 > N/P 20), while the retention time at the same cross-flow rate decreased slightly in the same order. These tendencies were more evident in the case of nanocomplexes formed with chitosan NAS-032-DNA compared to those comprising NAS-075 (*cf.* Figure 5a vs. Figure 5b, respectively). There are several possible explanations for this result. Firstly, a reduction in particle size with an increasing N/P ratio is expected to result in less scattered light for the smaller particles than the larger ones, as further elaborated below. Secondly, it might be that the net amount of formed nanocomplexes, which determines the MALS 90° signal intensity, could have decreased with the amount of chitosan in the system. Support for this explanation is provided below and in Figure 6 and Figure 7, which show the corresponding peaks registered with the UV signal detector. However, a third explanation is that as the amount of added chitosan increases with N/P ratio, the PECs become more hydrophobic, thus favouring a stronger interaction with the membrane thus leading to a lower recovery (Table S1). As it stands, it is likely that the observed differences in peak areas are the result of a combination of these three factors. Of note, in the case of the chitosan NAS-075-DNA nanocomplexes (Figure 5b), the magnitude of the peaks was smaller, and the observed trends are not as clear for the systems comprising chitosan NAS-032. It is challenging to interpret more broadly whether the observed discrepancies between these two types of nanocomplexes comprising different chitosans reflect the role of other factors such as the net charge compensation and stoichiometry, surface charge, hydrophobicity, or other conformational adaptation and rearrangements under kinetic control which might be at play. In this regards, previous studies focused on chitosan–dextran sulphate PECs have revealed the non-equilibrium nature of these systems when mixed spontaneously in contrast with those obtained by controlled dialysis [17].

It was also noted that the signal of particles eluting at the void peak was minimal in both cross-flow rates, which could be due to the increased focus time allowed in both cases to achieve better resolution. This result was in contrast to a previous study, which showed the presence of particles at the void peak decreased with the increasing cross-flow and increased separation force [66]. Additionally, the small void peak could result from a very small amount of unbound chitosan that elutes ahead of the nanocomplexes. In our study, in addition to the MALS and DLS detectors, we used two concentration detectors, namely a RI and UV-VIS one. The RI signal recorded was very noisy, as this detector is more suitable for completely dissolved samples. By contrast, the UV (λ = 280 nm) signal was much smoother than the RI one and hence was used as a non-destructive concentration detector, bearing in mind the caveat that its sensitivity can be limited for particles that range in the concentrations of few mg/L and lack specificity [67].

The regions of interest (ROI) of the fractograms were selected to evaluate the data and determine the radius of gyration (*R_g_*) and the hydrodynamic radius (*R_h_*) of the eluting fractions. *R_g_* was determined from the angular dependence of the MALS and UV (concentration) data. *R_g_*, also known as the root mean square (RMS) radius, describes the distribution of mass around the centre of gravity of the molecule. For a flexible random coil structure, *R_g_* is an average value of all the possible conformations. The hydrodynamic radius (*R_h_*) data in the ROI were also determined in the flow mode using an online DLS detector. 

The fractograms of the UV, along with the *R_g_* and *R_h_* traces of the nanocomplexes comprising NAS-032 and NAS-075 chitosans at different N/P ratios, are shown in Figure 6 and Figure 7. Here, *R_g_* and *R_h_* values were described across the full width at half maximum on the fractograms. Insight into the formed particles’ sizes and structural properties differences were gleaned from this analysis. As mentioned earlier, AF4 separation relies on the diffusion coefficient of the eluting species (Equation (1)); thus, complexes with larger sizes are bound to remain closer to the accumulation membrane for longer times and face reduced laminar flow in the channel. Therefore, the larger the particle, the longer its elution time [40]. We observed that the PECs eluted earlier at lower cross-flow rate (1 mL/min) as opposed to the higher cross-flow rate. This is the expected consequence of the rapid decline of the perpendicular force executed by the cross-flow at 1 mL/min contrary to the more gradual decline at 3 mL/min (Figure 1). The UV signal declined with the increasing N/P ratio for the nanocomplexes evidencing that the concentration of formed nanocomplexes decreased. AF4 results of PECs have been reported to show an increase in both *R_g_* and *R_h_* with elution time, due to an effective separation in normal mode [68]. A closer inspection of Figure 6 and Figure 7, shows that across the elution profile, the *R_g_* and *R_h_* data varied differentially for the various N/P ratios and cross-flow rate. Notably, the traces of both *R_g_* and *R_h_* climbed more steeply for NAS-032 nanocomplexes, particularly at N/P 5 charge ratio, than on the NAS-075 PECs. Additionally, the difference in both radii between the smallest and the largest eluting fractions was more pronounced at N/P 5 than at 20 and in the case of nanocomplexes comprising chitosan NAS-032-DNA (Figure 6) than those comprising chitosan NAS-075-DNA (Figure 7). These results are consistent with the notion that nanocomplexes formed with chitosan NAS-032 are more polydisperse in size than those obtained with chitosan NAS-075, and that the increasing charge ratios result in more monodisperse size distributions. The fractionation of nanocomplexes by AF4 and online detection by DLS analysis, can give a better estimate of size distribution polydispersity, in contrast to batch mode DLS measurements where the presence of a small number of aggregates can have an amplified effect on the PDI by scattering light more intensely [69]. Ma et al. used AF4 to determine the particle size and free fraction of polymer content from chitosan–DNA complexes by fluorescent labelling of chitosan with rhodamine B [34,35]. They observed that a significant amount of chitosan–rhodamine remained free and the polyplexes had broad size distributions at N/P ratio of 5. The elution profiles of NAS-075-DNA complexes at the cross-flow of 3 mL/min exhibited a broadening of the concentration peak and minimal *R_g_* and *R_h_* (closer to zero), as seen in Figure 7b,d,f. Figure S5 shows that the *R_g_* values of these nanocomplexes was zero until ~30–35 min of elution time (specifically for NP 5 and 10). We argue that these particles are too small (*R_g_* and *R_h_* < ~30 nm) to be detected by the MALS detector.

Light scattered from the small particles contribute to the angular dependency and their sum due to density fluctuations [70]. The intensity is described as a function of the scattering angle *θ*

(3) $$\frac{R_{\theta }\left(\theta \right)}{KC}=MP\left(\theta \right)$$

where *C* is the concentration, *K* is a constant dependent on the solvent–polymer system, *M* is molar mass, *R_θ_* is Rayleigh relation which is proportional to the intensity of scattered light, and $P\left(\theta \right)$ is known as the form factor describing the angular dependence of the scattered light and depends on the size (i.e., the radius of gyration, *R_g_*) and the shape of the particles. Here, the light scattered intensity, along with the concentration determined through UV detector, was used to calculate *R_g_.*

**Figure 8:**
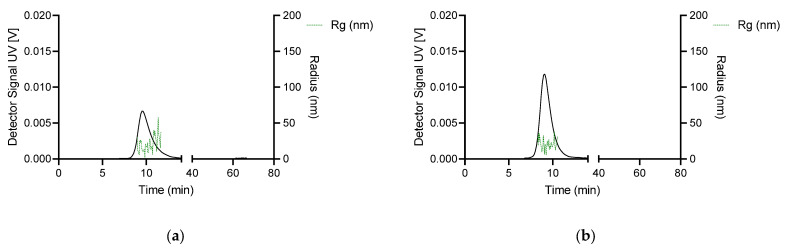
Fractograms for UV-Vis, *R_g_* of (**a**) deploy nas-032, and (**b**) deploy nas-075 DNA nanocomplexes at N/P charge ratio 20, obtained through asymmetric flow field-flow fractionation at cross-flow (XF) of 3 mL/min. The black trace is UV signal (concentration), the green dots are *R_g_*.

Once the parent chitosan–DNA PECs were characterised, we were also interested in comparing their properties with PECs formed by their depolymerised chitosan derivatives for AF4-MALS-UV analysis. In order to achieve better fractionation of these complexes, we doubled the injection volume and applied the cross-flow rate at 3 mL/min. Under such conditions we could not achieve a good elution profile for the complexes, except for those formed at N/P 20 (Figure 8). An inspection of the fractograms in Figure 8 shows that the oligomeric chitosan–DNA complexes start to elute significantly earlier than their parent counterparts at an equivalent crossflow rate, i.e., 3 mL/min. They started to elute within 9–12 min, indicative of the formation of small size assemblies with shorter retention time. The UV signal for deploy nas-032-DNA PEC was less intense than that of deploy nas-075-DNA PEC, denoting that the amount of PEC formed in the former case is lower than in the latter. The ascending trend of *R_g_* across the eluting ROI was not as pronounced as seen for the parent chitosan PECs at the similar N/P ratio 20 (*cf.* Figure 6f and Figure 7f). Also note the greater variability of the *R_g_* data, presumably due to the low concentration or size that may fall below the detection limit of the MALS detector. This is more clearly seen in the fractograms showing the corresponding MALS 90° and UV signals (Figure S6).

**Figure 9:**
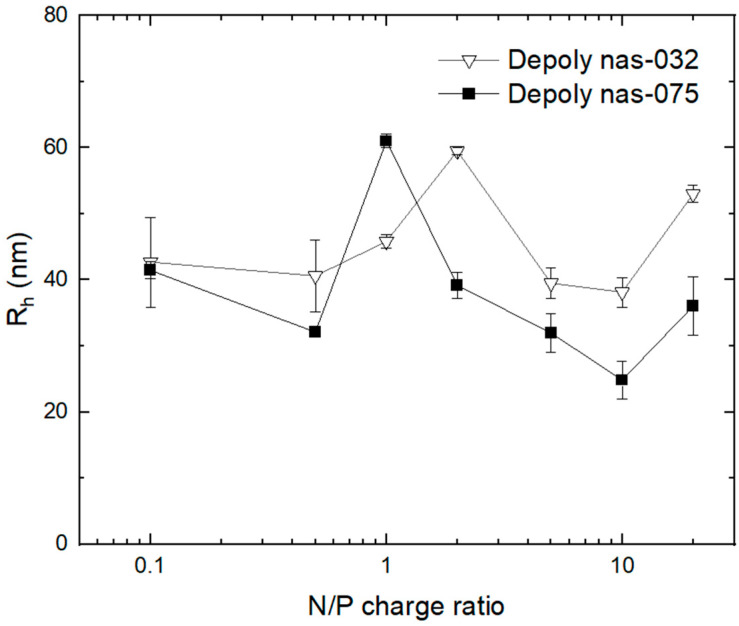
Hydrodynamic radius (*R_h_*) of depolymerised chitosan derivatives and calf-thymus DNA polyplexes prepared at various amine to phosphate (N/P) charge ratios determined through batch-mode NIBS-DLS.

Given the low detection resolution of flow-mode online DLS size analysis during AF4 observed for depolymerised chitosan–DNA polyplexes, we used batch-mode NIBS-DLS to determine the particle size distribution of these systems at varying N/P ratio, and the results are shown in Figure 9. 

An inspection of the data shows that as the N/P charge ratio increased due to the incremental concentration of chitosan, the polyplexes *R_h_* attained a maximal value at N/P ratios of ~1.0 and ~2.0 for deploy nas-075 and deploy nas-032, respectively. Zhang et al. [71] and Amaduzzdi et al. [72] reported a similar increase in the size of PECs close to the isoelectric point. Other studies [41] reported only slight variations in Rh in the range N/P 0.5 to 0.7, regardless of the degree of protonation of chitosans, suggesting that the degree of DNA compaction attained is similar. Beyond the isoelectric point, as the N/P ratio increased to 10, the size decreased to a minimum before rising again at N/P 20. These changes in size with N/P can be explained as the result of fluctuations in the conformation and structure of the PECs. As explained earlier, at the stoichiometric point, the ζ-potential of the complexes is close to zero (Figure 4) and the low electrostatic repulsion leads to aggregation. As the N/P ratio increases upon further chitosan addition, DNA can be expected to be condensed, hence the minimal *R_h_* being attained at N/P 10. Beyond this point, at the high N/P ration of 20, the adsorption of surplus chitosan at the surface of the PEC can explain the subsequent increase in size.

**Figure 10:**
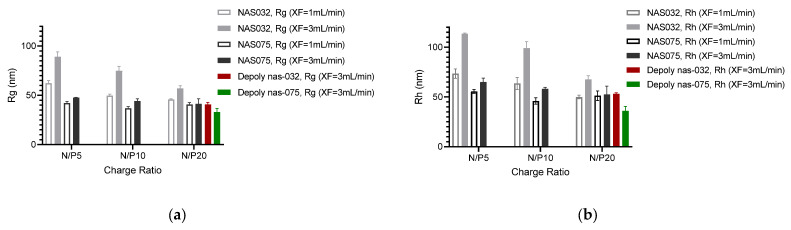
Radius of gyration (*R_g_*) (**a**) and hydrodynamic radius (*R_h_*) (**b**) of chitosan and DNA polyelectrolyte nanocomplexes and N/P ratios at different cross-flow rates.

The average radius of gyration (*R_g_*) values of the different polyplexes were also determined by flow online MALS AF4 across the eluting ROI and the results are summarised in Figure 10. Of note, *R_g_* values did not change drastically for nanocomplexes formed by chitosan NAS-075 for the different N/P charge ratios and at different cross-flow rates (Figure 10a). For NAS-032-DNA polyplexes, both radii decreased with increasing N/P charge ratio. The reduction in size with N/P charge ratio observed for the NAS-032-DNA PECs is consistent with the explanation offered above for the same trend seen in the PECs comprising depolymerised chitosans at N/P ratios beyond their stoichiometric charge compensation, the result of condensation of DNA. We can argue that the needed amount of added chitosan to achieve the same degree of compaction of DNA was invariably greater in the NAS-032 than in NAS-075. Earlier studies by Bordi et al. suggested that a large excess of polycation (chitosan) was required in order to achieve smaller, globular structures with a narrow-size distribution complexes [73], however this has not addressed the role of chitosan’s Mw and DA, which we offer in the present report. 

However, another possible explanation for the difference in size dependence of the nanocomplexes formed by the two parent chitosans may stem from the greater chain flexibility of the chitosan NAS-032 than the low DA chitosan NAS-075, in keeping with previous studies [74,75,76]. The greater chain flexibility in the high-DA chitosan NAS-032 might result in greater conformational disorder, yielding lower capacity to condense DNA. Moreover, it is expected that greater amounts of added chitosan further increase the enthalpic gain associated with DNA condensation. In addition to the DA for PEC formation, as stated above, another important parameter is the Mw of the chitosan. By comparing the sizes of the PECs at N/P 20, it is evident that the decrease in chitosan’s Mw accompanies the decrease in the size of the PECs (Figure S7). Note in Figure 10 that PECs formed by depolymerised chitosan at N/P 20 showed very little *Rg* variation suggestive of a compact structure. In the case of the PECs comprised by depolymerised chitosan, the *R_h_* was determined by NIBS-DLS in batch mode, in contrast with the parent chitosan PECs that were analysed in flow-mode by AF4. In this regard, it is interesting to note that the cross-flow rate also played a role in the size of the PECs. Invariably larger sizes were assessed at cross-flow rate 3 rather than at 1 mL/min.

Additionally, it should be noted that the difference of *R_g_* amid NAS-075 and deploy nas-075 PECs is less marked than that between NAS-032 and deploy nas-032 PECs. Interestingly, the deploy-032 chitosan PECs had similar *R_g_* and *R_h_* to NAS-075 chitosan PECs. This is a possible indication that the morphology of the PECs might have changed with Mw for the high DA chitosan. Novoa-Carballal et al. suggested that chitosan with intermediate degree of acetylation tends to adopt a more relaxed and flexible conformation, such as coiled shape [74]. This greater chain flexibility on the NAS-032 chitosan is expected to be lost upon deoplymerisation, thus resulting in a loss of conformational adaptation and the attainment of compact PEC structures of similar dimensions to those formed by the more charged parent and depolymerised chitosans of low DA.

AF4 analysis of *R_g_* and *R_h_* can also be harnessed to gain information regarding the internal structure, shape, and architecture of the nanocomplexes [77,78]. The rho ratio (*ρ* = *R_g_*/*R*_h_), also known as the shape factor, provides insight into the conformation and shape of macromolecules and nanoparticles [25]. 

The relationship between the *R_g_* and *R_h_* for a compact sphere is given by the following equation:

(4) $$R_{g}=\sqrt{\frac{3}{5}}R_{h}=0.775R_{h}$$

A shape factor of 0.775 to 1.000 corresponds to a homogenous to a hollow sphere, respectively, and *ρ* values greater than 1, are characteristic of a rod, random coil, and branched polymers [25,79]. For a given population, a constant shape factor through the elution is diagnostic of consistent conformations, where *R_g_* and *R_h_* both increase at the same rate with elution time. This is contrary to what we have observed in the chitosan–DNA nanocomplexes, in which *R_g_* and *R_h_* increased with varying rates and different among each other gradients with elution time. *R_h_* values were smaller than *R_g_* at lower elution time and then upsurged at a higher time (Figure 6 and Figure 7). This result points to a changing conformation with increasing size.

**Figure 11:**
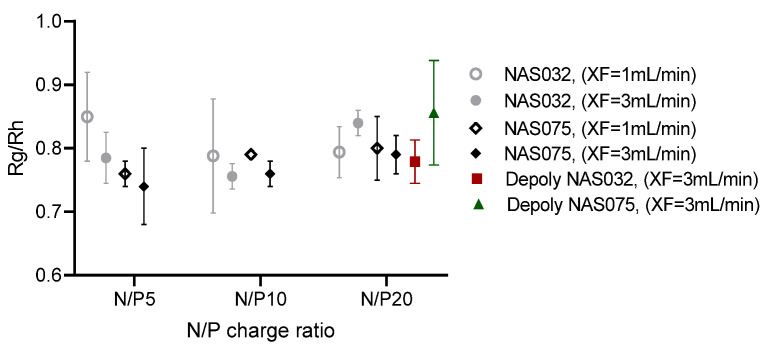
Graph representing average *R_g_*/*R_h_* (shape factor, *ρ*) values of chitosan (NAS-032, NAS-075, deploy nas-032, and deploy nas-075)–DNA nanocomplexes at different N/P charge ratios and cross-flows.

As previously mentioned, the fitting model used was a random coil, but we also noted that at lower elution times (i.e., smaller nanocomplexes) the sphere model also showed a suitable fitting (Figure S8). This fact further indicates that the nanocomplexes might adopt varying shapes while eluting at different elution times. The average shape factor *ρ* of the nanocomplexes ranged from 0.74–0.85, as summarised in Figure 11, corresponds closely with the theoretical value for a homogeneous compact spherical conformation (*ρ* = 0.775). The relatively narrow range of the *ρ* values indicates that the different elution profiles do not significantly affect the conformation of the nanocomplex particles. Indeed, the *ρ* values lie between those for homogeneous hard solid spheres (*ρ* = 0.775) and that of a hollow sphere (*ρ* = 1) [79]. The value of *ρ* changed from 0.84 to 0.779 in the case of complexes formed from NAS-032 polymer and deploy nas-032, respectively, and is consistent with our suggestion that a more compact spherical is attained for the low Mw chitosan PEC. Notably, NAS-075 and deploy nas-032 present a very similar *ρ* at the same cross-flow rate. This is also concurrent with the hypothesis that these systems adopt a similar compact spherical conformation, which is not influenced by chitosan’s DA. It is worth noting that the ~3-fold greater amount of deploy nas-032 than deploy nas-075 was used to form the PECs at N/P 20. Ma et al. [35] also described the conformation of chitosan–DNA polyplexes using AF4/UV-Vis/MALS+DLS. They reported *ρ* values in the range 1.0–1.5 of complexes, suggesting a polymeric star and cluster conformation rather than the compact spherical morphology observed in our work. Of note, the chitosans used in their work (Mn 10–76 kDa and DA 8–28%) had characteristics that spanned those of sample NAS-075 (Mn ~54 kDa DA ~4.7%) used in our study. We have now included a chitosan of very high DA, namely NAS-032 (Mn ~92 kDA, DA ~48%) and its depolymerised chitosan (Mn ~5 kDa, DA ~48%), that extends the DA range of the series of Ma et al. The conformation of the PECs obtained in our study varied substantially from those of Ma et al. [35], as evidenced from the measured *ρ* values. We can only speculate that the discrepancy between both studies may stem from the fact that in our work, we did not label chitosan with rhodamine, as well as in other subtle differences in the methodology to prepare the PECs. Bravo-Anaya et al. [41] studied the physicochemical aspects of chitosan–DNA complexation for a series of fully characterised polymeric chitosans (Mw ~75–540 kDa, DA 3–61%). They concluded that the DA and Mw had little influence on the complex formation and stoichiometry when the net charge of chitosan was correctly accounted for. Previous studies, using tapping-mode atomic force microscopy (AFM), reported that the complexation of both linear and circular pDNA with chitosan (Mw 162 kDa and DA 10%) yields a mixture of toroidal and rod-like structures [58]. In their quantitative analysis, they have also shown that the ratio of polyplexes in the form of toroids and rods decreases with the increasing degree of acetylation, thus indicating that the charge density of the cation plays an important role in determining shape of the DNA-chitosan complex [58]. In agreement with our results, Liu et al. investigated the physicochemical properties of chitosan and DNA polyelectrolyte complexes and found that the morphology of the polyplexes is strongly influenced by the N/P charge ratios. At lower N/P ratio, the DNA is not completely entrapped, however at higher charge ratios, the polyplexes without any free DNA evolve to form a spherical shape [80]. MacLaughlin et al. reported toroids and rod-shaped particles when an 8 kDa chitosan condenses a plasmid with a diameter of 66 nm [57], unlike here, where even the PECs comprising low Mw chitosan seem to form spherical complexes. 

In turn, Kim et al. reviewed the morphology and mechanism of complexation of nucleic acid-based PECs [81]. They state that N/P~1 (i.e., stoichiometric point) complexes are neutral and form a macroscopic drop (due to phase separation). The morphology of virus-like particles is of extreme importance as it entails important features of their biological behaviour [62].

Moreover, for complexes formed at N/P ~1, intercomplex disproportionation dominates, while if N/P is further away from 1, intracomplex disportionation does, thus leading to a tadpole conformation [81], in agreement with the proposal made in previous studies on nanocomplexes comprising a high-DA chitosan [72]. By contrast, in our case, the nanocomplexes formed at N/P charge ratios far from the isoelectric point (N/P 5, 10 and 20) showed average shape factor values that correspond to a spherical morphology, despite similarities in Mw with previous studies [27] and no apparent influence of the DA over the wide range in our two samples (DA ~5–48%). Only further studies addressing these aspects in further detail will shed further insight into the role of the varying factors known to be at play, primarily the structural characteristics of chitosan and DNA, and the N/P charge ratio.

### 3.5. Negative Stain Transmission Electron Microscopy (TEM)

**Figure 12:**
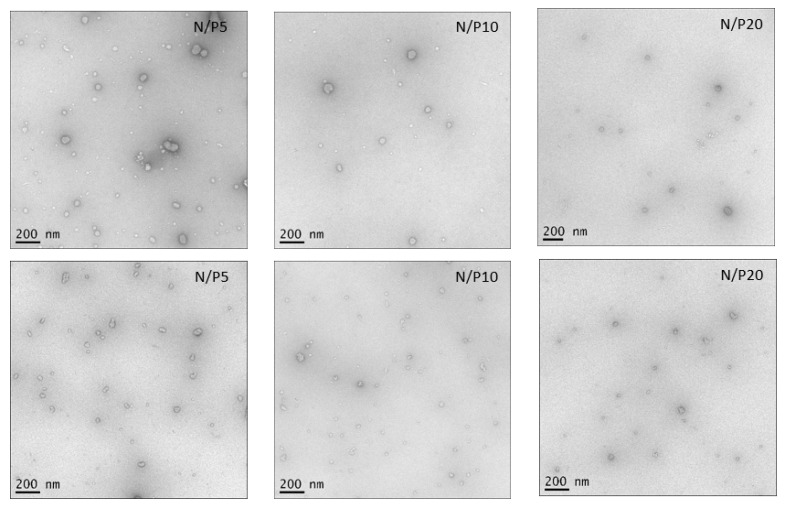
Negative stain transmission electron micrographs of chitosan–DNA polyelectrolyte complexes. Upper panel) NAS-032-DNA complexes; Lower panel) NAS-075-DNA complexes. Magnification is shown in bars.

Further evidence on the size morphology of the parent chitosan–DNA nanocomplexes was gained by TEM visualisation (Figure 12). The chitosan–DNA nanocomplexes exhibited a blend of particles ranging from spherical to non-spherical shapes. It was seen that the variation in shape was more evident for lower charge ratios. Micrographs of nanocomplexes formed with NAS-032 at N/P ratio 5 showed a mixture of globular and elongated structures. The size and number of the polyplexes declined with the increasing charge ratio, with most of the complexes exhibiting sizes < ~200 nm. This result generally agrees with the AF4 results, even when the visualised structures deviated somewhat from homogenous spherical structures. We could not perform TEM images for the PECs assembled from the depolymerised chitosans. However, based on the AF4 data, we can assume them to also be spherical in shape. Of note, the mismatch in morphology observed between the TEM images and AF4 data can stem from the fact that TEM imaging captures the bulk sample rather than the fractionated species achieved by AF4. The results obtained from microscopic techniques like TEM or AFM provide data of qualitative nature, and only a limited number of objects are visualised in the image field of view (which might be unrepresentative of the whole sample), along with changes that ensue during sample preparation (e.g., aggregation) [82]. Thus, using AF4 coupled with multi-detection, we argue here, enables a quantitative and overall robust analysis the sample’s size and shape distributions.

## 4. Conclusions

AF4 with multi-detection (MALS, DLS, and UV) was used to fractionate and characterise the particle size distribution of chitosan and DNA nanocomplexes regarding the radius of gyration and hydrodynamic radius, thus enabling them to determine the shape factor and infer on their morphology. Two fully characterised high-purity chitosans of Mw (110–160 kDa) and their depolymerised derivatives Mw (6–14 kDa) and broadly different degrees of acetylation (0–48%) were used to obtain nanocomplexes of N/P molar charge ratios 5, 10, and 20. The findings reported here shed new light into the role of the DA of chitosan on the compaction of DNA at these N/P ratios, where the high DA chitosan, due to its greater chain flexibility, results in more expanded structures at low N/P ratio and greater dependency of the size on N/P. In turn, the low DA chitosan forms more compact structures with little dependency on N/P ratio, in agreement with previous studies [41]. We analysed the influence of two different cross-flow rates on the AF4 elution profiles and found that for our nanocomplexes, both methods were successful for the quantitative characterisation and the determination of their size and shape. The cross-flow rate did not significantly impact the separation. We found that the complexation behaviour for chitosan–DNA polyplex formation is independent from the Mw for chitosans of low DA, and the PECs formed by this chitosan attain a comparable size and shape, thus suggesting that the polymers and depolymerised oligomers interact with DNA in a similar manner. Our results expand upon previous AF4 studies to characterise chitosan–DNA complexes [34,35] and allowed us to gain insight on the morphology of the complexes that concurred with TEM imaging evidence of the presence of predominantly spherical structures. These results contrast from previous studies [34,55] that have claimed the formation of toroidal, rod-like, and tadpole objects. Only further comparative studies using different techniques such as small-angle X-ray scattering (SAXS) and Cryo-TEM will help to elucidate the origin of these discrepancies and give an additional insight on the structure, size, and shape of these polyplexes and resolve their distribution profiles. The ability of AF4 to separate particles from the aggregates of chitosan polymers underscores the robustness of this technique and its accuracy in contrast to conventional techniques such as SEC. Our study contributes to the existing knowledge on chitosan–DNA self-assembling and to expanding the range of techniques to address their size and morphology. The insights gained from this study will be of assistance in understanding the role of these aspects on the biological performance of these systems as non-viral gene delivery nanocarriers. 

## Figures and Tables

**Table 1:** Details the conditions of two methods used to fractionate and characterise chitosan/DNA polyelectrolyte complexes by AF4.

Flow Setup	Method 1			Method 2		
**Injection flow rate (mL/min)**	0.2			0.2		
**Injection/Focus time (min)**	9 @ flow rate 1.80 mL/min			6 @ flow rate 3.30 mL/min		
**Transition time (min)**	1			0.2		
**Cross-flow rate (mL/min)**	1.0			3.0		
**Elution steps**	Time (min)	Flow rate (mL/min)	Type	Time (min)	Flow rate (mL/min)	Type
	10	0.4	Exponent: 0.4	0.2	3	Constant
	20	0.15	Exponent: 0.8	30	3	Exponent: 0.4
	15	0.05	Linear: 1	5	0.22	Exponent: 0.8
	15	0.05	Constant: 0.8	5	0.11	Exponent: 0.8
				20	0.06	Constant
**Rinse time (min)**	0.5			0.5		
**Total run time (min)**	70.5			66.9		

**Table 2:** Degree of acetylation of chitosans determined using UV spectrophotometry (DA*) and corresponding values provided by the manufacturer (DA**).

Chitosan Sample	DA* (%)	DA** (%)
**NAS-032**	43.4	48.0
**NAS-075**	4.7	0

**Table 3:** Table listing the details of the molecular weight distributions of NAS-032 and NAS-075 and their derivatives, i.e., deploy nas-032 and deploy nas-075 determined through SEC and AF4.

Chitosan Sample	Technique	Mn (g/mol)	Mw (g/mol)	Mz (g/mol)	Ð (=Mw/Mn)	Recovery (%)
**NAS-032**	SEC	(9.18 ± 0.68) · 10^4^	(1.64 ± 0.01) · 10^5^	(3.01 ± 0.00) · 10^5^	1.79 ± 0.11	88.1
	AF4	(9.19 ± 0.79) · 10^4^	(1.42 ± 0.02) · 10^5^	(2.08 ± 0.11) · 10^5^	1.55 ± 0.11	30.0
**Deploy nas-032**	SEC	(5.41 ± 3.09) · 10^3^	(1.02 ± 0.21) · 10^4^	(1.38 ± 0.14) · 10^4^	2.26 ± 0.97	51.0
	AF4	(8.22 ± 0.49) · 10^3^	(8.39 ± 0.50) · 10^3^	(8.57 ± 0.52) · 10^3^	1.02 ± 0.001	25.7
**NAS-075**	SEC	(5.47 ± 0.50) · 10^4^	(1.18 ± 0.06) · 10^5^	(2.88 ± 0.19) · 10^5^	2.17 ± 0.29	93.5
	AF4	(6.42 ± 0.35) · 10^4^	(1.23 ± 0.05) · 10^5^	(2.49 ± 0.71) · 10^5^	1.92 ± 0.17	38.7
**Deploy nas-075**	SEC	(1.95 ± 1.51) · 10^3^	(6.12 ± 0.68) · 10^3^	(1.03 ± 0.31) · 10^4^	4.68 ± 3.98	73
	AF4	(1.42 ± 0.23) · 10^4^	(1.46 ± 0.22) · 10^4^	(1.51 ± 0.20) · 10^4^	1.02 ± 0.01	18.1

## Data Availability

The data presented in this study are openly available in: Sajid, Ayesha; Castronovo, Matteo and Goycoolea, Francisco M. On the Fractionation and Physicochemical Characterisation of Self-Assembled Chitosan–DNA Polyelectrolyte Complexes Data Set. University of Leeds [Dataset] https://doi.org/10.5518/1340.

## Supplementary Materials

The following supporting information can be downloaded at: https://www.mdpi.com/article/10.3390/polym15092115/s1, Figure S1: Chromatograph representing the overlay of light scattering signal (MALS 90°) for parent chitosan (NAS-032 and NAS-075) and their depolymerized derivatives (deploy nas-032 and deploy nas-075). Figure S2: SEC chromatographs for NAS-032 and NAS-075 presenting Molar Mass (Da) and radius (nm). Figure S3: Conformation plots (log-*R_g_* vs. log-M) for chitosans NAS-032 and NAS-075. Figure S4: SEC refractive index signals for NAS-032, NAS-075 and mobile phase 0.3 M acetic acid/0.2 M sodium acetate, pH = 3.7. Figure S5: Fractograms of UV-Vis, *R_g_* and *R_h_* of NAS075-DNA nanocomplexes (from left to right N/P 5, 10 and 20) at cross-flow 3 mL/min. Figure S6: Fractograms of UV-Vis and MALS 90° of polyelectrolyte complexes formed with deploy nas-032 and deploy nas-075 at N/P charge ratio = 20 eluted at cross-flow of 3 mL/min. Figure S7: The effect of molecular weight of chitosan on the hydrodynamic radius (*R_h_*) of chitosan-DNA polyelectrolyte complexes formed at N/P charge ratio of 20. Figure S8: Elugram of NAS-032-DNA complex at the N/P = 5. Table S1: AF4 mass recovery percentage of chitosan-DNA nanocomplexes at different cross-flow rates and using chitosan of varying degree of acetylation.
