# Supplementary material for: On the Fractionation and Physicochemical Characterisation of Self-Assembled Chitosan–DNA Polyelectrolyte Complexes
Source: Polymers (Basel). 2023 Apr 28;15(9):2115. doi: 10.3390/polym15092115 (PMC10180698; doi:10.3390/polym15092115)
Supplement: Supplementary file 1 [file polymers-15-02115-s001.zip › polymers-2100839-SI.pdf]

Supplementary Information

On the fractionation and physicochemical characterisation of self-assembled chitosan and DNA polyelectrolyte complexes

Ayesha Sajid, Matteo Castronovo and Francisco M Goycoolea*

School of Food Science and Nutrition, University of Leeds, Leeds LS2 9JT, United Kingdom
F.M.Goycoolea@leeds.ac.uk

*Correspondence: F.M.Goycoolea@leeds.ac.uk

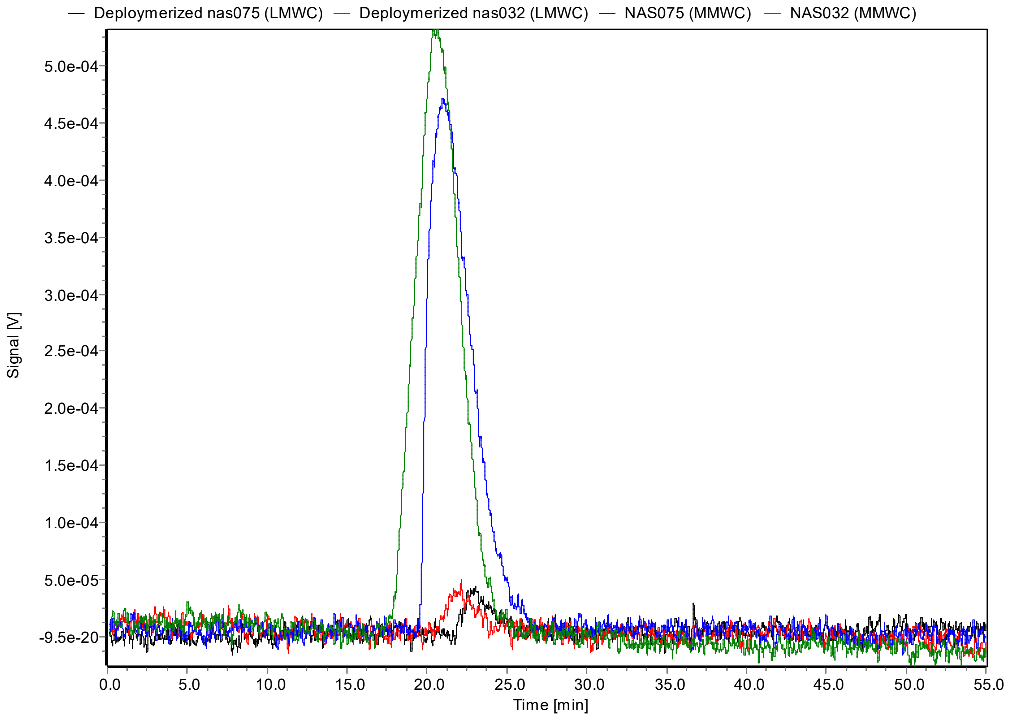

Figure S1: SEC chromatogram showing overlay of light scattering signal (MALS 90°) for the parent chitosan (NAS-032 and NAS-075) and their depolymerized derivatives (deploy nas-032 and deploy nas-075). The reduction in the signal intensity for the low molecular weight chitosan samples can be seen.

Figure S2 (a) and (c) represents the molar mass of chitosan sample with respect to the retention time and the concentration for NAS-032 and NAS-075 respectively. The molar mass of chitosan decreased at higher retention time as larger particles elute first. Similarly, the radius of the polymer also decreased S2 (b) and (d)

NAS-032

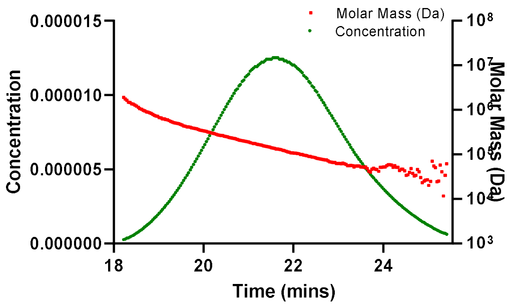

(a)

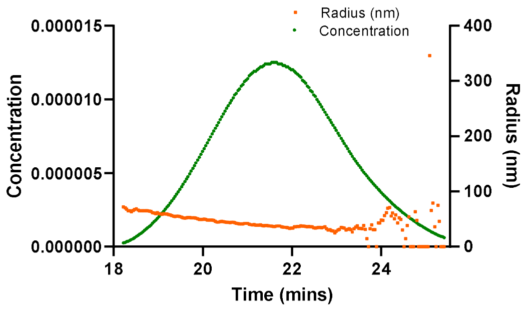

(b)

NAS-075

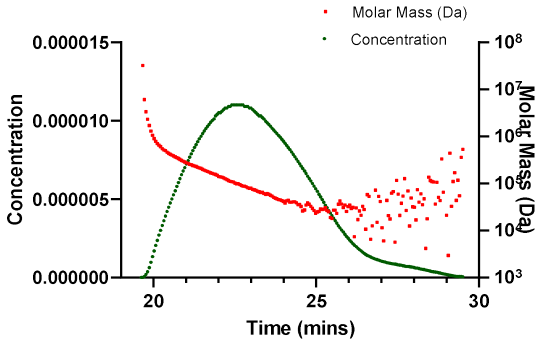

(c)

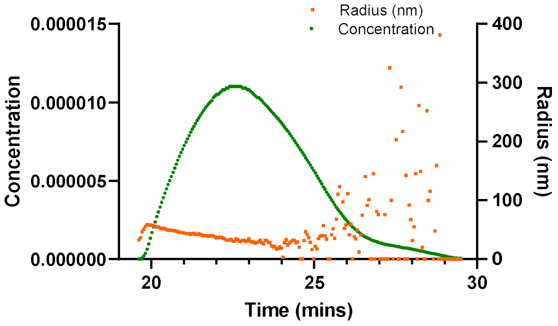

(d)

Figure S2: SEC chromatographs for NAS-032 (top) and NAS-075 (bottom). Green signal corresponds to the concentration through DRI detector; red is the molar mass (Da) and orange is the radius of gyration (nm)

Figure S3 below shows the conformation Flory plots for the chitosan samples. The plot of the log R vs log M is almost linear with a slope for NAS-032=0.216 ±0.02 (a) and that of NAS-075=0.181±0.07 which can give an idea about the conformation of the polymer. Weinhold et al. (2011) [1], studied the conformation of chitosan in reference to its viscosity and stated that slope can differ from 0 (compact sphere) to 0.6-0.8 (random coil) to very stiff chains. In this study, the slope for NAS-032 was significantly deviated from zero and could form a more flexible/random coil, also indicated by the large Mw of NAS-032 (164 KDa) as compared to NAS-075 (118 KDa). The value of slope for NAS-075 is very close to zero and could form a compact structure.

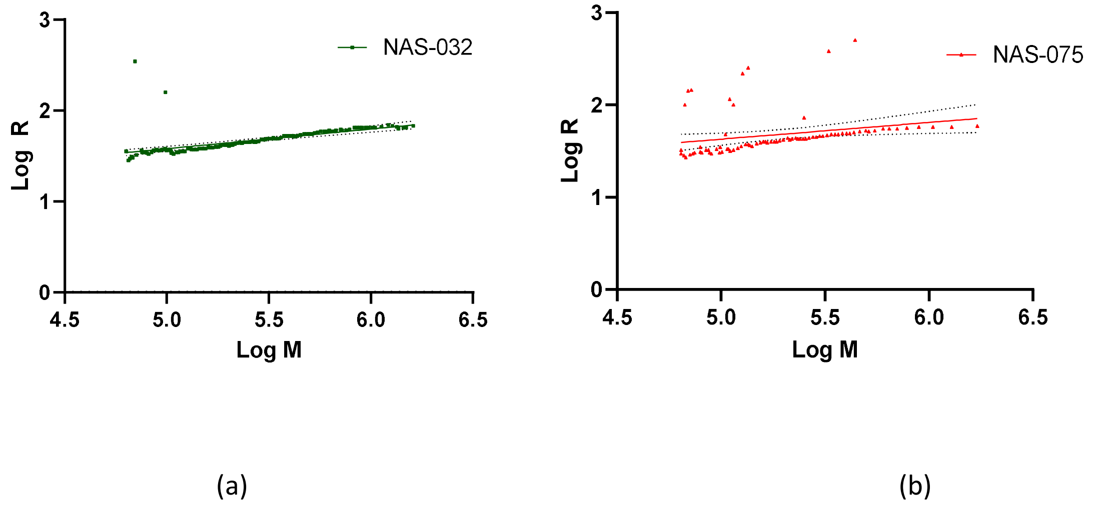

Figure S3: Conformation plots (log-Rg vs log-M) for chitosans (a) NAS-032 and (b) NAS-075

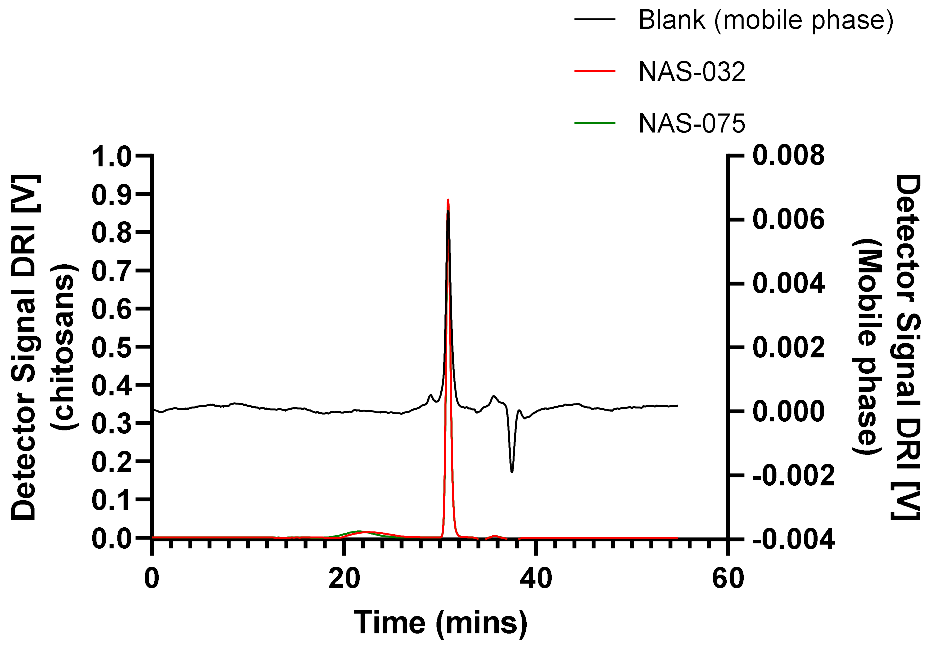

Figure S4: SEC refractive index signals for NAS-032 (green), NAS-075 (red) and and mobile phase 0.3M acetic acid/0.2M sodium acetate, pH=3.7 (black).

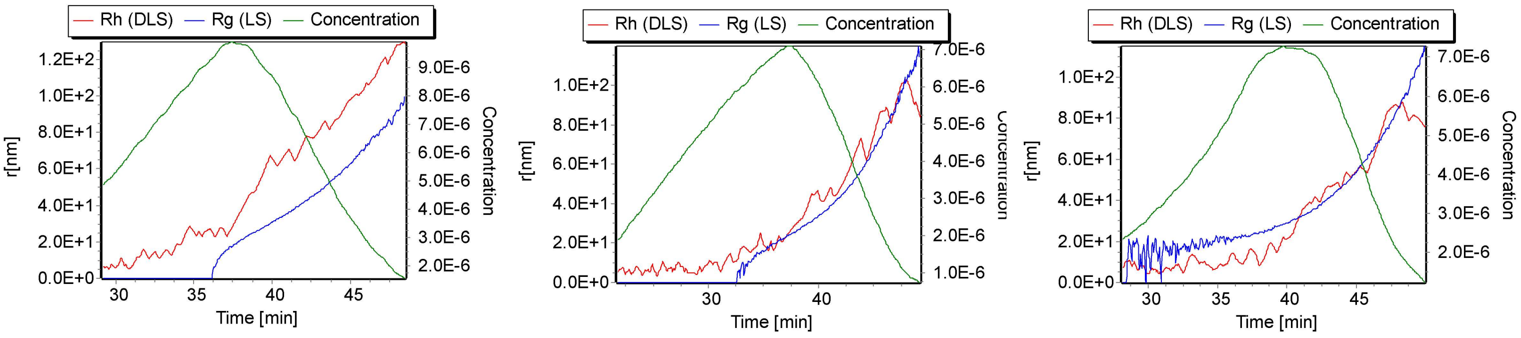

Figure S5: Fractograms of UV-Vis, R_g and *Rh* of NAS075-DNA nanocomplexes (from left to right N/P 5, 10 and 20) at cross-flow 3 mL/min.

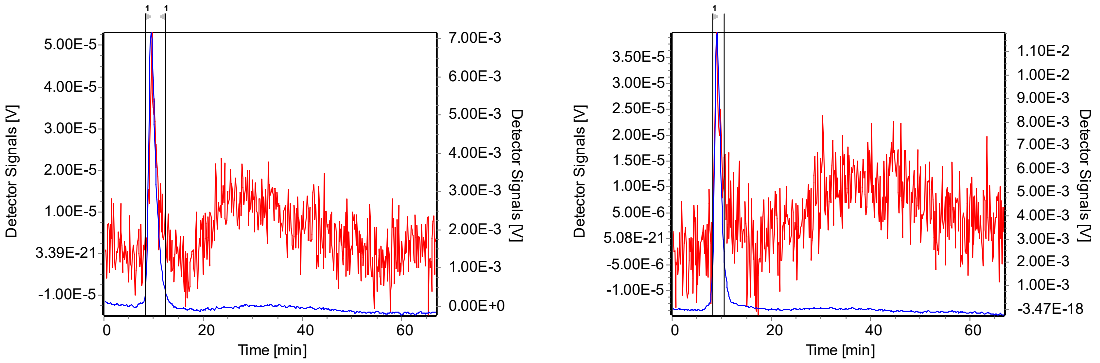

(a) (b)

Figure S6: Fractograms obtained from AF4-MALS-UV-vis of polyelectrolyte complexes formed of a) deploy nas-032 and b) depoly nas-075 chitosan and ctDNA at N/P charge ratio 20 eluted at the cross-flow of 3mL/min. The blue trace is the UV-vis signal and red trace is MALS 90° signal. The small size PEC elutes very early (between 9-11 mins) and we see some unresolved complexes or aggregrates appreaing as a small shoulder on right but with very little concentration. The noisy light scattering signal can be due to intensity fluctuations caused by multiple sized popolations.

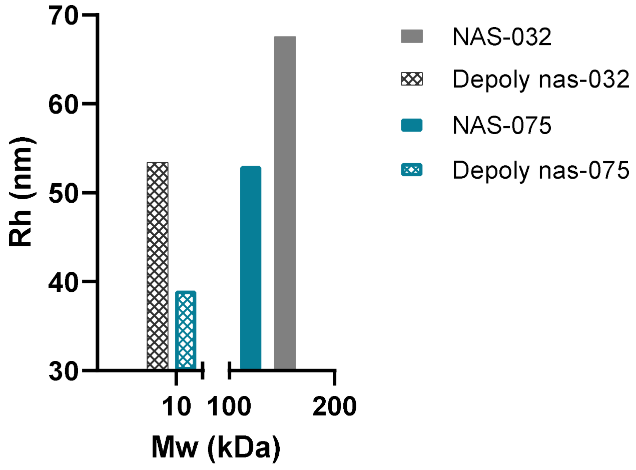

Figure S7: The effect of molecular weight of chitosan on the hydrodynamic radius (R_h) of chitosan-DNA polyelectrolyte complexes formed at N/P charge ratio=20. The R_h for depoly nas-032 and deploy nas-075 PEC was determined through batch-mode NIBS-DLS while for NAS-032 and NAS-075 PEC the R_h was obtained from online DLS (flow mode) attached to AF4, cross-flow=3mL/min

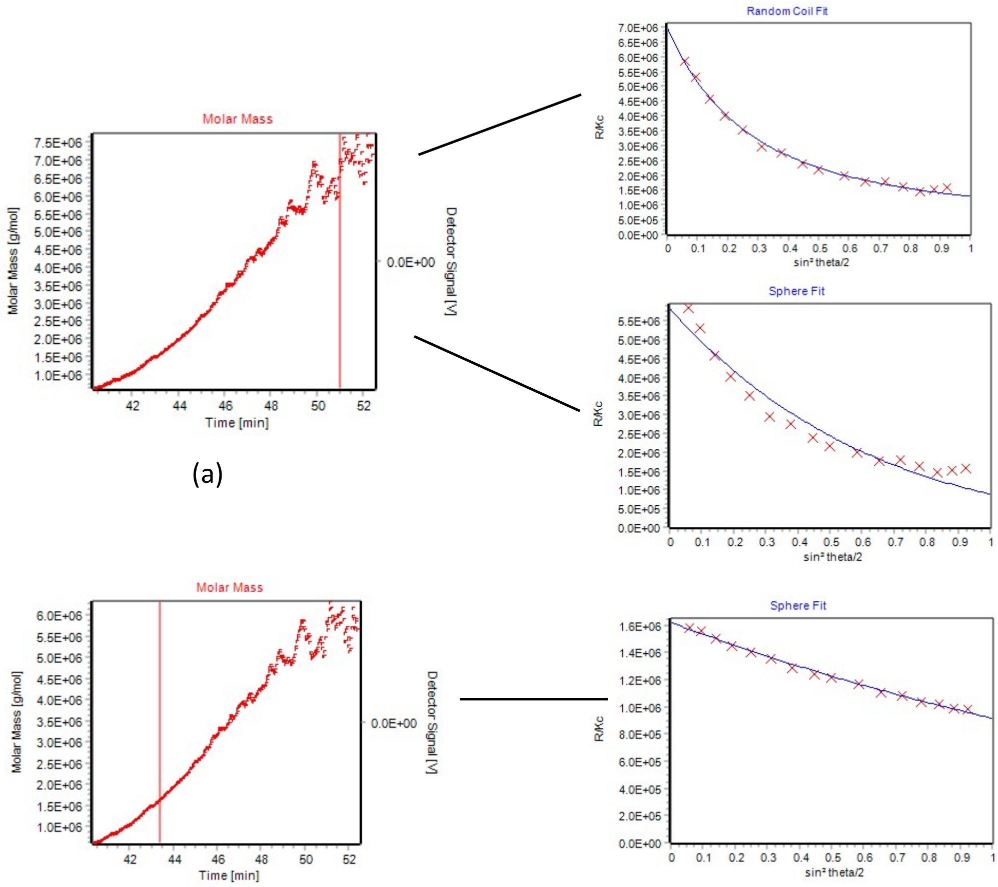

(b)

Figure S8: Elugram of NAS-032-DNA complex at the N/P=5. It was observed that at higher elution time (a) the complexes showed a better fit to the random coil model, while at lower elution times the sphere model also showed a good correlation. This could mean that smaller particles eluting first tend to be more spherical than the larger particles eluting after them, supporting our argument that the mixed population of chitosan-DNA nanocomplexes have a predominantly spherical conformation.

Table S1: AF4 mass recovery percentage of chitosan-DNA nanocomplexes at different cross-flow rates and using chitosan of varying degree of acetylation.

**N/P Charge ratio**	**Mass recovery %**			
	**NAS-032**		**NAS-075**	
	Cross-flow 3 mL/min	Cross-flow 1 mL/min	Cross-flow 3 mL/min	Cross-flow 1 mL/min
**5**	50.18	67.84	57.05	68.14
**10**	43.53	60.55	52.09	65.80
**20**	27.85	35.41	49.04	63.27

References

[1] Weinhold M.X. and Thöming J.(2011) *On conformational analysis of chitosan*. Carbohydrate Polymers. 84, 1237–1243
